# Prevalence, incidence and correlates of low risk HPV infection and anogenital warts in a cohort of women living with HIV in Burkina Faso and South Africa

**DOI:** 10.1371/journal.pone.0196018

**Published:** 2018-05-01

**Authors:** Admire Chikandiwa, Helen Kelly, Bernard Sawadogo, Jean Ngou, Pedro T. Pisa, Lorna Gibson, Marie-Noelle Didelot, Nicolas Meda, Helen A. Weiss, Michel Segondy, Philippe Mayaud, Sinead Delany-Moretlwe, on behalf of the HARP Study Group

**Affiliations:** 1 Wits Reproductive Health and HIV Institute, Faculty of Health Sciences, University of the Witwatersrand, Johannesburg, South Africa; 2 Clinical Research Department, London School of Hygiene & Tropical Medicine, London, United Kingdom; 3 Centre de Recherche Internationale pour la Santé, Université de Ouagadougou, Ouagadougou, Burkina Faso; 4 INSERM, EFS, University of Montpellier, Montpellier, France; Istituto Nazionale Tumori IRCCS Fondazione Pascale, ITALY

## Abstract

**Objective:**

To report the prevalence and incidence of low-risk human papillomavirus infection (LR-HPV) and anogenital warts (AGW) among women living with HIV (WLHIV) in Burkina Faso (BF) and South Africa (SA), and to explore HIV-related factors associated with these outcomes.

**Methods:**

We enrolled 1238 WLHIV (BF = 615; SA = 623) aged 25–50 years and followed them at three time points (6, 12 and 16 months) after enrolment. Presence of AGW was assessed during gynaecological examination. Cervico-vaginal swabs for enrolment and month 16 follow-up visits were tested for HPV infection by Inno-LiPA® genotyping. Logistic regression was used to assess risk factors for prevalent infection or AGW. Cox regression was used to assess risk factors for incident AGW.

**Results:**

Women in SA were more likely than those in BF to have prevalent LR-HPV infection (BF: 27.1% vs. SA: 40.9%; p<0.001) and incident LR-HPV infection (BF: 25.8% vs. SA: 31.6%, p = 0.05). Prevalence of persistent LR-HPV was similar in the two countries (BF: 33.3% vs. SA: 30.4%; p = 0.54), as were prevalence and incidence of AGW (Prevalence: BF: 7.5% vs. SA: 5.7%; p = 0.21; Incidence: BF: 2.47 vs. SA: 2.33 per 100 person-years; p = 0.41). HPV6 was associated with incident AGW (BF: adjusted Hazard Ratio (aHR) = 4.88; 95%CI: 1.36–17.45; SA: aHR = 5.02; 95%CI: 1.40–17.99). Prevalent LR-HPV (BF: adjusted Odds Ratio [aOR = 1.86]; 95%CI: 1.01–3.41; SA: aOR = 1.75; 95%CI: 0.88–3.48); persistent LR-HPV (BF: aOR = 1.92; 95%CI: 0.44–8.44; SA: aOR = 2.81; 95%CI: 1.07–7.41) and prevalent AGW (BF: aOR = 1.53; 95%CI: 0.61–3.87; SA: aOR = 4.11; 95%CI: 1.20–14.10) were each associated with low CD4+ counts (i.e. <200 vs. >500 cells/μL). Duration of ART and HIV plasma viral load were not associated with any LR-HPV infection or AGW outcomes.

**Conclusion:**

LR-HPV infection and AGW are common in WLHIV in sub-Saharan Africa. Type-specific HPV vaccines and effective ART with immunological reconstitution could reduce the burden of AGW in this population.

## Introduction

Anogenital human papillomavirus (HPV) infection is the most frequent sexually transmitted infection (STI) globally.[1] Low Risk (LR) HPV types 6 and 11 are the predominant causes of anogenital warts (AGW), a source of psychosocial distress,[2] and physical discomfort (including bleeding and itching).[3] Approximately 25% of AGW spontaneously regress,[3] but recurrence is common, resulting in high medical costs from repeated treatment.[4]

There is little literature on the epidemiology of LR-HPV infection and AGW in Sub-Saharan Africa. Regional variations in LR-HPV prevalence varies from 2% in Western Africa, 4% in Southern Africa and 8% in Eastern Africa.[5] Similary, the prevalence of AGW ranges from 4–11% in Western, 2–14% in Southern and 3–11% in Eastern African regions.[6] The natural history of HPV infection is altered by HIV infection, and HPV prevalence is higher where HIV is more common or among individuals with HIV infection.[7] People living with HIV who have AGW tend to experience florid and prolonged clinical manifestations of AGW because of their impaired immune response.[8] Low CD4+ counts (<200 cells/μL) are associated with an increased risk of AGW.[9, 10] The impact of antiretroviral therapy (ART) on the prevalence and incidence of AGW is unclear, with conflicting findings from different studies. Some report that ART reduces incidence,[10] while other studies have shown that it does not reduce incidence.[7, 11]

Three vaccines against HPV have been licensed,[12] two of which (quadrivalent and nonavalent) have activity against HPV types 6 and 11 that cause 90% of AGW.[13, 14] These vaccines have been steadily introduced into national immunization programmes in both high and low and middle income countries (LMIC), although the pace of introduction has been slower in LMIC.[15] Data from countries which have included the quadrivalent vaccine (against HPV6/11/16/18 types) in their vaccination programme show significant declines in the number of cases of AGW among vaccinated women and some herd-immunity effect in unvaccinated heterosexual men.[16] Decisions about which vaccine to include in a vaccination programme are driven by cost-effectiveness considerations. Since a high-burden of AGW is associated with significant costs to the health system in LMIC and this is worsened by high HIV prevalence, data on the prevalence, incidence and correlates of LR-HPV and AGW is important for informing vaccine programme decision-making and resource allocation.[12] To address this question, we analysed data from a cohort of women living with HIV (WLHIV) who participated in the HPV in Africa Research Partnership (HARP) study of cervical cancer screening in Burkina Faso and South Africa.

## Materials and methods

### Study design, population and sample collection

We conducted a prospective cohort study among WLHIV aged 25–50 years and residing in Ouagadougou, Burkina Faso (BF) and Johannesburg, South Africa (SA) between December 2011 and October 2012. A full description of the study design and procedures has been published elsewhere.[17] Briefly, enrolled participants were followed-up every 6 months up to 18 months. Data on clinical, socio-demographic and behavioural characteristics were collected by interviewer-administered questionnaire at each visit. The presence of AGW was assessed at enrolment and months 6, 12 and endline by trained nurse midwives during standardised pelvic examination. Cervical samples were collected using a Digene cervical sampler (Qiagen, Courtaboeuf, France) for HPV-DNA testing and genotyping, a swab from the ecto/endocervix to detect cervical sexually transmitted infections (STIs) by molecular methods and a vaginal smear to diagnose bacterial vaginosis and *Candida albicans* by Gram stain at both enrolment and endline visits. Blood samples were collected at enrolment to confirm HIV-1 serostatus, perform Herpes Simplex Virus-2 (HSV-2) and syphilis serologies as well as obtain baseline HIV-1 plasma viral load (PVL) measurement and CD4+ T-lymphocytes counts. The monitoring of CD4+ counts was done at subsequent 6-monthly visits.

### Laboratory methods

HIV-1 serostatus was diagnosed using two rapid tests according to national guidelines.[18, 19] Testing for CD4+ cells was performed using FACScount (Becton-Dickinson, NJ). Plasma HIV-1 RNA was assessed using real-time PCR (Abbott RT HIV-1) in BF and COBAS Taqman (Roche Diagnostics) in SA, with a lower limit of detection of 40 copies/ml in both countries. Laboratories subscribed to international external quality assessment schemes such as the UK-NEQAS for CD4+ counts[20] and QCMD for HIV-1 PVL testing.[21]

HSV-2 serology was performed using the Kalon® gG2 ELISA (Kalon Diagnostics, UK) and syphilis serology by a combination of a *Treponema pallidum* haemagglutination (TPHA) and rapid plasma reagin (RPR; BioMérieux, Lyon, France in BF and Immutrep carbon antigen RPR, Omega Diagnostics in SA). *Neisseria gonorrhoeae*, *Chlamydia trachomatis*, *Mycoplasma genitalium* and *Trichomonas vaginalis* were detected using nucleic acid amplification tests, the Sacace simplex assays (Sacace, Como, Italy) in BF and the APTIMA Combo (Gen-Probe, San Diego, CA) in SA. The Nugent’s score was used for vaginal flora reading of Gram-stained vaginal smears, with diagnosis of bacterial vaginosis made for scores ≥7, and examined for presence of *Candida*.

HPV DNA testing and genotyping was performed at the virology laboratory of University of Montpellier (UM) using INNO-LiPA HPV Genotyping Extra Assay (Innogenetics, Courtaboeuf, France). The assay is based on the amplification of a 65-bp fragment in the L1 gene and detects 28 HPV types, including all the high-risk or probable high-risk types (16, 18, 26, 31, 33, 35, 39, 45, 51, 52, 53, 56, 58, 59, 66, 68, 73, 82) as well as the following LR HPV genotypes (6, 11, 40, 43, 44, 54, 69/71, 70,74).[22]

### Statistical analysis

Any LR-HPV type infection was defined as detection of *≥*1 of the 9 genotypes described above. LR-HPV type-specific persistence was defined as being positive for the same type at enrolment and endline. Type-specific LR-HPV incidence was defined as the proportion of women who were negative for a specific LR type at enrolment and positive for that type at endline. Prevalent AGW were defined as the presence of clinically-defined *condyloma acuminata* at the enrolment visit. Incident AGW were defined as the first documented occurrence of *condyloma acuminata* in a woman previously documented not to have AGW. Time to incident AGW was calculated from date of enrolment visit to the date of first AGW detection.

Descriptive statistics were used to summarize the prevalence of any LR-HPV infection and AGW. For bivariate analysis, socio-demographic, sexual behaviour, presence of other laboratory detected STI (as part of the screening process at enrolment), clinical signs and HIV-related risk factors were chosen for assessment *a priori* as known correlates for HPV and AGW acquisition. As LR-HPV prevalence was common, associations with exposure variables were estimated from prevalence ratios obtained from logistic regression using marginal standardization to estimate prevalence ratios, and the delta method to estimate 95% confidence intervals (CIs). Associations between LR-HPV persistence and exposure variables were estimated with generalized estimating equations to account for multiple LR-HPV infection and multiple infection states (persistence and clearance).[23] To explore associations of any LR-HPV and AGW with HIV-related factors, pre-specified analyses included stratification by site, ART use, duration on ART (≤ or >2 years), HIV-1 viral suppression (< or ≥1000 copies/ml) and CD4+ cell counts at enrolment. Logistic regression (Model 1) was used to identify independent associations between any prevalent LR-HPV or AGW and HIV-related factors, after adjustment for potential confounders (non HIV-related factors) that were associated with the outcomes in bivariate analysis at p<0.10.[24] A second logistic regression model (Model 2) adjusted for CD4+ cell count at enrolment in addition to factors adjusted for in Model 1 was run to explore associations with ART duration and HIV-1 viral suppression. Cox regression was performed to analyse predictors of incident AGW, with and without adjustment for potential confounders using the same model-building approach as described above under logistic regression. Data were analysed using Stata version 14 (Stata Statistical Software, College Station. TX: Stata Corporation).

### Ethics statement

Study protocols were approved by the Ministry of Health in Burkina Faso (no. 2012-12-089), the University of the Witwatersrand in South Africa (no. 110707), and the London School of Hygiene and Tropical Medicine (no. 7400). All participants provided written informed consent.

## Results

### Study population

Of the 1473 women screened, 1238 (615 in BF and 623 in SA) were enrolled. The median age of participants was 36 (interquartile range [IQR], 31–42) years in BF and 34 (IQR, 30–40) years in SA. At enrolment, two-thirds of the participants were on ART (BF: 68.6% in BF; 65.2% in SA) and this is because enrolment was stratified in the ratio of 2 ART users: 1 non-ART user. In BF, the median CD4+ count was 417 (IQR, 315–606) cells/μL among ART-naive participants and 446 (IQR, 309–600) cells/μL among those on ART. In SA, the median CD4+ count was 448 (IQR, 353–614) cells/μL among ART-naive and 420 (IQR, 279–567) cells/μL among those on ART. A more detailed description of study participants has been published elsewhere.[17]

### Prevalence and correlates of LR-HPV infection

**Fig 1:**
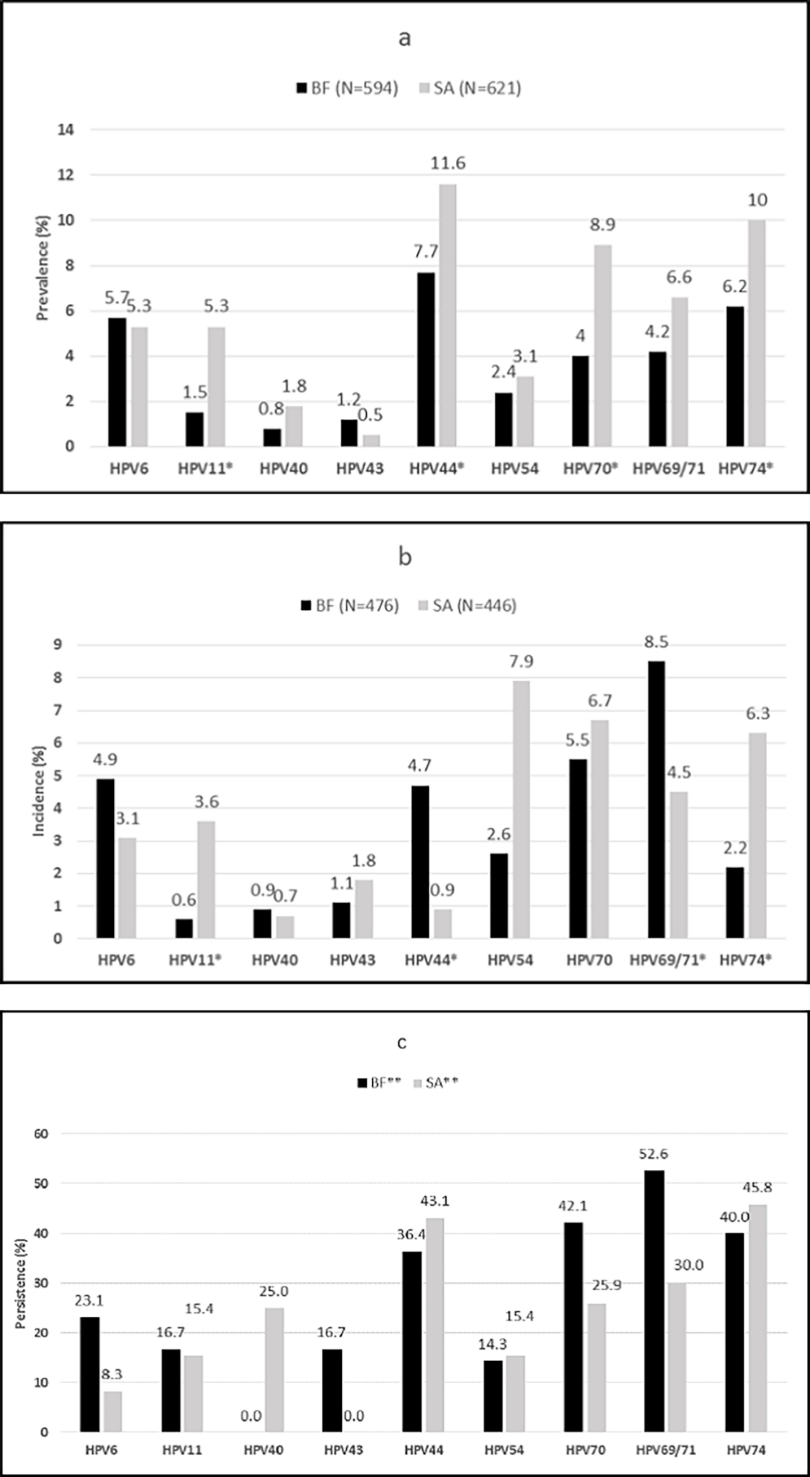
Type specific LR-HPV infection. (A) Prevalence at enrolment. (B) Incidence over 16 months. (C) Persistence over 16 months. *Significantly different between the two countries (i.e. p-value <0.05). **Persistence as a proportion of those positive for that specific type at enrolment.

Almost all participants (n = 1215; 98.1%) had valid HPV genotyping results [BF = 594 (96.6%); SA = 621 (99.7%)]. The prevalence of any LR-HPV was lower in BF (27.1%) than in SA (40.9%) (p<0.001), as were the prevalence of the following LR types: 11 (1.5% vs. 5.3%, p<0.001), 44 (7.7% vs. 11.6%, p = 0.02), 70 (4.0% vs. 8.9%, p = 0.001) and 74 (6.2% vs. 10.0%, p = 0.02). Type 6 prevalence was similar in both countries (BF 5.7% vs. SA 5.3%). Type 44 was the most prevalent LR-HPV type, for both countries (Fig 1A). In both countries, LR-HPV prevalence was lower among women who have had pregnancies compared to those who have been never pregnant (BF: adjusted Prevalence Ratio (aPR) = 0.54; 95%CI: 0.29–0.99 vs. SA: aPR = 0.62; 95%CI: 0.44–0.89). In BF alcohol use (aPR = 1.41; 95%CI: 1.01–1.97) and self-reported consistent condom use (aPR = 1.93; 95%CI: 1.07–3.47) were associated with higher LR-HPV prevalence. In SA LR-HPV prevalence was higher among women without a regular sexual partner compared to those with a regular partner (aPR = 2.39; 95%CI: 1.37–4.19) (Table 1).

**Table 1:** The prevalence and correlates of LR-HPV^a^ infection among 594 WLHIV in BF and 621 in SA.

	Burkina Faso, N = 594					South Africa, N = 621				
		LR positive	Crude	Multivariate^b^			LR positive	Crude	Multivariate^b^	
	N	n (%)	PR (95% CI)	aPR (95% CI)	p-value	N	n (%)	PR (95% CI)	aPR (95% CI)	p-value
**Partner status**										
Single or unmarried	305	101 (33.2)	1	1		357	146 (40.9)	1	1	
Married or cohabiting	289	60 (20.8)	0.62 (0.47–0.82)	0.62 (0.33–1.19)	0.15	264	108 (40.9)	1.00 (0.83–1.21)	1.26 (0.69–2.32)	0.45
**Alcohol use**										
Never	395	94 (23.8)	1	1		432	179 (41.4)	1	1	
Ever	199	67 (33.7)	**1.41 (1.09–1.84)**	**1.41 (1.01–1.97)**	0.05*	189	75 (39.7)	0.96 (0.78–1.18)	1.03 (0.80–1.33)	0.81
**Number of pregnancies**										
0	24	11 (45.8)	1	1		31	18 (58.1)	1	1	
1–2	200	63 (31.5)	0.69 (0.43–1.11)	0.76 (0.41–1.40)	0.38	329	136 (41.3)	**0.71 (0.51–0.99)**	**0.64 (0.46–0.90)**	0.01*
3–4	370	87 (23.5)	**0.51 (0.32–0.82)**	**0.54 (0.29–0.99)**	0.05*	261	100 (38.3)	**0.66 (0.47–0.92)**	**0.62 (0.44–0.89)**	0.01*
**Condom use**										
Never	68	10 (14.7)	1	1		31	11 (35.5)	1	1	
Sometimes	70	10 (14.3)	0.97 (0.43–2.18)	0.82 (0.38–1.76)	0.61	172	63 (36.6)	1.03 (0.62–1.73)	1.26 (0.70–2.29)	0.44
Always	162	63 (38.9)	**2.64 (1.45–4.84)**	**1.93 (1.07–3.47)**	0.03*	303	133 (43.9)	1.24 (0.76–2.02)	1.35 (0.76–2.40)	0.31
**Currently has regular sexual partner**										
Yes, cohabiting	196	42 (21.4)	1	1		258	105 (40.7)	1	1	
Yes, non-cohabiting	105	40 (38.1)	1.78 (1.24–2.56)	0.83 (0.22–3.12)	0.79	243	99 (40.7)	1.00 (0.81–1.24)	1.35 (0.73–2.52)	0.34
No	72	21 (29.2)	1.36 (0.87–2.14)	-		11	8 (72.7)	**1.79 (1.21–2.64)**	**2.39 (1.37–4.19)**	0.01*
**Lifetime number of sex partners**										
1	138	28 (20.3)	1	1		17	10 (58.8)	1	1	
2–4	394	109 (27.7)	1.36 (0.94–1.97)	0.97 (0.60–1.57)	0.92	283	109 (38.5)	**0.65 (0.43–1.00)**	**0.61 (0.39–0.94)**	0.03*
5+	62	24 (38.7)	1.91 (1.21–3.01)	0.70 (0.34–1.44)	0.34	227	98 (43.2)	0.73 (0.48–1.12)	0.65 (0.41–1.01)	0.06
**Candida albicans**										
No	491	141 (28.7)	1	1		558	223 (40.0)	1	1	
Yes	83	14 (16.9)	0.59 (0.36–0.97)	0.50 (0.24–1.03)	0.60	52	25 (48.1)	1.20 (0.89–1.63)	1.14 (0.80–1.62)	0.47

^a^: LR-HPV = HPV6/11/40/43/44/54/70/69/71/74

^b^: Adjusted Prevalence Ratio (aPR) from logistic regression using marginal standardisation

*All factors with p<0.05 are in bold

### Associations between prevalent LR-HPV infection and HIV-related factors

Of the 1215 women with valid HPV results, 32 (2.6%) reported that they were not on ART at enrolment despite undetectable HIV-1 PVL (BF = 24; SA = 8). Given the uncertainty of their ART status, these women were excluded from HIV-related factors analyses. Further analyses were conducted on the remaining 1183 participants (BF = 570; SA = 613) to determine the effect of HIV-related factors on any LR-HPV prevalence (Table 2). In both countries, LR-HPV prevalence was higher among women with a low CD4+ count (<200 cells/μL vs. >500 cells/μL; Model 1 BF: adjusted Odds Ratio (aOR) = 1.37; 95%CI: 1.13–1.65 vs. Model 1 SA: aOR = 1.15; 95%CI: 0.99–1.33) (Table 2). Among ART users, a low CD4+ count was also associated with a higher risk of prevalent LR-HPV infection (Model 1 BF: aOR = 1.41; 95%CI: 0.89–2.24 vs. Model 1 SA: aOR = 1.58; 95%CI: 1.06–2.34). The duration on ART (i.e. >2 years vs. ≤2 years on ART) and HIV-1 PVL at enrolment were not significantly associated with prevalent LR-HPV infection (Table 2).

**Table 2:** Associations between prevalent LR-HPV^a^ and HIV-related factors among 570 WLHIV in BF and 613 in SA.

			Burkina Faso^b^				South Africa^c^		
			N = 570^d^				N = 613^d^		
	Description	N	n (%)	Model 1	Model 2	N	n (%)	Model 1	Model 2
				aOR (95% CI)	aOR (95% CI)			aOR (95% CI)	aOR (95% CI
	ART >2 years	194	52 (26.8)	1.00	1.00	225	84 (37.3)	1.00	1.00
**ART Status**	ART ≤2 years	218	67 (30.7)	1.24 (0.92–1.67)	1.19 (0.88–1.60)	179	84 (46.9)	1.22 (0.94–1.57)	1.15 (0.87–1.50)
	ART-naive	158	36 (22.8)	0.87 (0.60–1.26)	0.82 (0.57–1.18)	209	83 (39.7)	1.11 (0.87–1.43)	1.11 (0.86–1.42)
	>500	217	52 (24.0)	1.00		225	82 (36.4)	1.00	
**Enrolment**	351–500	161	39 (24.2)	1.05 (0.87–1.25)		183	74 (40.4)	1.04 (0.92–1.17)	
**CD4+ count**	201–350	125	40 (32.0)	1.11 (0.92–1.33)		148	66 (44.6)	1.09 (0.98–1.23)	
**(cells/μL)**	<200	66	24 (36.4)	1.37 (1.13–1.65)*		57	29 (50.9)	1.15 (0.99–1.33)	
	**Enrolment CD4+ count (cells/μL)**								
	>500	52	10 (19.2)	1.00		87	35 (40.2)	1.00	
**ART-naive**	351–500	42	7 (16.7)	0.86 (0.35–2.15)		70	24 (34.3)	1.10 (0.74–1.64)	
	201–350	45	12 (26.7)	1.37 (0.63–2.97)		46	22 (47.8)	1.12 (0.72–1.73)	
	<200	19	7 (36.8)	1.43 (0.55–3.71)		6	2 (33.3)	0.99 (0.35–2.77)	
	**HIV-1 viral suppression**								
	<1000 copies/ml	328	96 (29.3)	1.00	1.00	324	132 (40.7)	1.00	1.00
	≥1000 copies/ml	55	18 (32.7)	1.13 (0.75–1.69)	0.98 (0.62–1.56)	76	35 (46.1)	1.14 (0.84–1.54)	1.09 (0.80–1.48)
	**HIV-1 viral detection**								
	≤40 copies/ml	286	82 (28.7)	1.00	1.00	135	54 (40.0)	1.00	1.00
**ART users**	>40 copies/ml	97	32 (33.0)	1.03 (0.85–1.24)	0.93 (0.75–1.16)	265	113 (42.6)	0.92 (0.81–1.03)	0.91 (0.81–1.03)
	**Enrolment CD4+ count (cells/μL)**								
	>500	165	42 (25.5)	1.00		138	47 (34.1)	1.00	
	351–500	119	32 (26.9)	1.09 (0.74–1.60)		113	50 (44.3)	1.28 (0.91–1.80)	
	201–350	80	28 (35.0)	1.37 (0.93–2.02)		102	44 (43.1)	1.24 (0.87–1.76)	
	<200	47	17 (36.2)	1.41 (0.89–2.24)		51	27 (52.9)	1.58 (1.06–2.34)*	

^a^Any LR-HPV = HPV6/11/40/43/44/54/70/69/71/74; Adjusted Odds Ratio (aOR): Model 1

^b^In Burkina Faso, associations with LR-HPV were adjusted for alcohol use, condom use and number of pregnancies

^c^In SA, associations with LR-HPV were adjusted for regular sex partner, number of pregnancies and number of lifetime sex partners; For Model 2: same adjustment as Model 1, in addition to enrolment CD4+ cell count

^d^Overall, 32 participants (BF: 24; SA: 8) reported that they were not on ART at enrolment despite undetectable HIV-1 viral load. Given the uncertainty of their ART status, these were excluded from the analyses

*All factors with p<0.05 are in bold.

### Incidence and persistence of LR-HPV infection and effect of HIV-related factors

Overall, 963/1238 (77.8%) WLHIV attended the endline visit at a median 16 (IQR, 15.6–16.8) months and the majority (922) had HPV-genotyping results at both enrolment and endline (BF = 476; SA = 446). Of these, 264 (28.7%) had incident LR-HPV of any type during follow-up. The incidence of any LR-HPV infection was lower in BF compared to SA (BF: 25.8% vs. SA: 31.6%, p = 0.05). This was also true for most individual types (11, 54 and 74) with the exception of types 44 (BF: 4.7% vs. SA: 0.9%, p = 0.01) and 69/71 (BF: 8.5% vs. SA: 4.5%, p = 0.02) which were significantly higher in BF compared to SA (Fig 1B).

A third of WLHIV (310/922) were defined as having persistent LR-HPV infection; the total number of persistent infections was 396 (BF = 156; SA = 240). The frequency of persistent infection of any LR-HPV at endline was similar in both countries (BF: 33.3% vs. SA: 30.4%; p = 0.54). Persistence for individual types was largely similar between the two countries (Fig 1C). A low CD4+ count (i.e. <200 cells/μL vs. >500 cells/μL) was associated with a higher likelihood of having persistent LR-HPV infection. The association was stronger among ART users (Model 1 BF: aOR = 3.21; 95%CI: 0.90–11.40 vs. Model 1 SA: aOR = 3.33; 95%CI: 0.96–11.55). The duration on ART at enrolment and HIV-1 PVL were not significantly associated with persistent LR HPV infection (S1 Table).

### Prevalent AGW and effect of prevalent LR-HPV infection and HIV-related factors

Overall, 81 (6.7%) WLHIV had AGW detected at enrolment; the prevalence did not vary by country (BF: 7.5% vs. SA: 5.7%; p = 0.21). In univariate analysis (data not shown), prevalent AGW were associated with age, number of partners in the last 3 months before enrolment, presence of genital ulcers for both countries and being HSV-2 seropositive in BF or having bacterial vaginosis in SA. Infection with any LR-HPV type, multiple LR-HPV infections, and infection with types 6 (Model 2 BF aOR = 3.14; 95%CI: 1.11–9.01 and SA aOR = 1.79; 95%CI: 0.49–6.51) and 11 (Model 2 BF aOR = 20.12; 95%CI: 4.06–99.71 and SA aOR = 7.90; 95%CI: 3.11–20.09) were significantly associated with prevalent AGW (Table 3). In BF, HPV type 44 infection (Model 2 aOR = 3.59; 95%CI: 1.53–8.39), was strongly associated with prevalent AGW. Similary in SA, HPV type 74 infection (Model 2 aOR = 3.28; 95%CI: 1.35–7.98) was also significantly associated with prevalent AGW. AGW prevalence was higher among those with low CD4+ counts (i.e. <200 cells/μL vs. >500 cells/μL), particularly in SA among all women (aOR = 4.11; 95%CI: 1.20–14.10) and among ART users only (aOR = 3.93; 95%CI: 1.01–15.28). AGW prevalence was higher among those who had been on ART for two years or less compared to those on ART for more than two years at enrolment in both BF (Model 1 aOR = 1.78; 95%CI: 0.87–3.64) and SA (Model 1 aOR = 1.88; 95%CI: 0.80–4.38), although the association was less pronounced when adjusted for CD4+ count (Table 3).

**Table 3:** Effect of HIV-related factors and LR-HPV on AGW prevalence at enrolment among 572 WLHIV in BF and 613 in SA.

				Burkina Faso^a^				South Africa^b^	
				N = 572^c^				N = 613^c^	
	Description	N	n (%)	Model 1	aOR (95% CI)	N	n (%)	Model 1	Model 2
				aOR (95% CI)	Model 2			aOR (95% CI)	aOR (95% CI)
	Any LR-HPV^d^	155	22 (14.2)	2.80 (1.48–5.27)*	2.75 (1.44–5.23)*	251	21 (8.4)	2.14 (1.05–4.36)*	2.06 (1.01–4.21)*
	Multiple LR-HPV	34	9 (26.5)	6.50 (2.57–16.44)*	5.97 (2.49–14.30)*	65	10 (15.4)	4.65 (1.96–11.37)*	4.50 (1.81–11.19)*
	HPV6	33	5 (15.2)	3.21 (1.12–9.15)*	3.14 (1.11–9.01)*	33	3 (9.1)	1.62 (0.45–5.82)	1.79 (0.49–6.51)
	HPV11	9	5 (55.6)	21.54 (4.47–103.82)*	20.12 (4.06–99.71)*	33	9 (27.3)	7.32 (2.98–18.00)*	7.90 (3.11–20.09)*
	HPV40	5	0 (0.0)			11	1 (9.1)	1.79 (0.21–15.00)	1.48 (0.17–12.75)
**LR-HPV**	HPV43	7	2 (28.6)	4.08 (0.67–24.80)	4.03 (0.69–23.69)	3	0 (0.0)		
	HPV44	45	9 (20.0)	3.73 (1.61–8.67)*	3.59 (1.53–8.39)*	71	6 (8.5)	1.55 (0.60–4.00)	1.42 (0.54–3.72)
	HPV54	14	1 (7.1)	0.72 (0.09–6.10)	0.60 (0.07–5.44)	20	3 (15.8)	3.60 (0.93–13.83)	3.47 (0.85–14.15)
	HPV70	22	2 (9.1)	1.00 (0.22–4.63)	0.99 (0.21–4.57)	55	3 (5.5)	0.87 (0.25–3.02)	0.81 (0.23–2.84)
	HPV69/71^e^	25	4 (16.0)	1.65 (0.50–5.49)	1.57 (0.45–5.43)	40	2 (5.o)	0.83 (0.18–3.81)	0.81 (0.17–3.804)
	HPV74	35	5 (14.3)	2.10 (0.72–6.08)	2.19 (0.75–6.48)	61	8 (13.1)	3.34 (1.39–8.04)*	3.28 (1.35–7.98)*
	ART >2 years	236	16 (6.8)	1	1	226	10 (4.4)	1	1
**ART status**	ART ≤2 years	173	20 (11.6)	1.78 (0.87–3.64)	1.78 (0.82–3.58)	178	15 (8.4)	1.88 (0.80–4.38)	1.41 (0.57–3.50)
	ART-naive	163	9 (5.5)	0.63 (0.25–1.58)	0.62 (0.25–1.59)	209	10 (4.8)	0.83 (0.32–2.11)	0.80 (0.31–2.05)
	>500	216	17 (7.9)	1		225	7 (3.1)	1	
**Enrolment**	351–500	162	10 (6.2)	0.70 (0.31–1.61)		183	11 (6.0)	22.5 (0.83–6.09)	
**CD4+ count**	201–350	123	9 (7.3)	0.91 (0.39–2.14)		148	12 (8.1)	2.92 (1.09–7.79)*	
**(cells/μL)**	<200	64	9 (14.1)	1.53 (0.61–3.87)		57	5 (8.8)	4.11 (1.20–14.10)*	
	**Enrolment CD4+ count (cells/μL)**								
	>500	52	0 (0.0)			87	2 (2.3)	1	
**ART-naive**	351–500	45	1 (2.2)	1		70	6 (8.6)	4.19 (0.77–22.80)	
	201–350	46	4 (8.7)	4.19 (0.45–39.00)		46	2 (4.4)	2.06 (0.27–15.54)	
	<200	19	4 (21.1)	11.7 (1.21–11.38)*		6	0 (0.0)		
	**HIV-1 viral suppression**								
	<1000 copies/ml	357	34 (10)	1	1	328	19 (6)	1	1
	≥1000 copies/ml	52	2 (4)	0.36 (0.08–1.61)	0.41 (0.09–1.92)	71	6 (9)	1.18 (0.44–3.17)	0.89 (0.31–2.52)
	**HIV-1 viral detection**								
	≤40 copies/ml	315	25 (8)	1	1	139	7 (5)	1	1
**ART users**	>40 copies/ml	94	11 (12)	1.36 (0.62–2.98)	1.60 (0.70–3.68)	260	18 (7)	1.15 (0.46–2.92)	1.09 (0.43–2.79)
	**Enrolment CD4+ count (cells/μL)**								
	>500	164	17 (10.4	1		138	5 (3.6)	1	
	351–500	117	9 (7.7)	0.72 (0.31–1.68)		113	5 (4.4)	1.30 (0.35–4.36)	
	201–350	77	5 (6.5)	0.60 (0.21–1.69)		102	10 (9.8)	2.66 (0.85–8.38)	
	<200	45	5 (11.1)	1.08 (0.38–3.11)		51	5 (9.8)	3.93 (1.01–15.28)*	

Adjusted Odds Ratio (aOR): For Model 1

^a^In BF, associations with AGW were adjusted for age, number of partners in last 3 months, presence of genital ulcers and HSV2

^b^In SA, associations with AGW were adjusted for age, number of partners in last 3 months, presence of genital ulcers and bacterial vaginosis; For Model 2: same adjustment as Model 1, in addition to enrolment CD4+ count

^c^Overall 30 participants (BF: 22; SA: 8) reported that they were not on ART at enrolment despite undetectable HIV-1 viral load. Given the uncertainty of their ART status, these were excluded from the analyses

^d^Any LR-HPV = HPV6/11/40/43/44/54/70/69/71/74

^e^INNOLiPA does not discriminate between HPV types 69 and 71

*All factors with p<0.05 are in bold.

### AGW incidence and association with LR HPV infection and HIV-related factors

A total of 32 new AGW events (BF = 16; SA = 16) were recorded for the 992 (BF = 468; SA = 524) WLHIV who were followed up for a total of 1390 person-years. The overall AGW incidence was 2.30 per 100 person-years (BF: 2.47 vs. SA: 2.33 per 100 person-years, p = 0.41). In univariate analysis (data not shown), incident AGW were associated with age for both countries and number of partners in the last 3 months before enrolment in BF or having mycoplasma genitalium in SA. Infection with any LR-HPV at enrolment was associated with having an incident AGW; this association was statistically significant in BF (Model 1 adjusted Hazard Ratio [aHR] = 5.78; 95%CI: 2.13–16.22) (Table 4). Prevalent type 6 infection at enrolment was associated with an almost 5-fold higher odds of incident AGW in both BF (Model 1 aHR = 4.88; 95%CI: 1.36–17.45) and SA (Model 1 aHR = 5.02; 95%CI: 1.40–17.99). In BF, prevalent HPV 74 infection was also significantly associated with incident AGW (Model 1 aHR = 6.02; 95%CI: 1.90–19.04). The incidence of AGW was higher in women with lower CD4+ counts, with an inverse dose-response relationship in SA; but the duration on ART at enrolment and HIV-1 PVL enrolment were not significantly associated with AGW incidence, even after adjustment for CD4+ count (Model 2).

**Table 4:** Effect of HIV-related factors and LR-HPV infection on incident AGW among 468 WLHIV in BF and 524 in SA.

		Burkina Faso^a^			South Africa^b^		
	Description	Number of	Model 1	Model 2	Number of	Model 1	Model 2
		events/Person	aHR (95% CI)	aHR (95% CI)	events/Person	aHR (95% CI)	aHR (95% CI)
		Years			Years		
**LR HPV**	Any LR-HPV^c^	10/158	5.78 (2.13–16.22)*	6.03 (2.15–16.89)*	8/270	1.77 (0.66–4.74)	1.74 (0.64–4.72)
	Multiple LR-HPV	1/20	7.38 (1.46–37.38)*	6.56 (1.27–38.81)*	2/64	2.22 (0.46–10.66)	2.09 (0.44–9.99)
	HPV6	3/34	4.88 (1.36–17.45)*	4.48 (1.25–16.09)*	3/36	5.02 (1.40–17.99)*	5.37 (1.47–19.70)*
	HPV11^d^	0/4			0/29		
	HPV40^d^	0/4			0/11		
	HPV43	1/7	6.63 (0.82–53.63)	6.80 (0.84–55.07)	0/4		
	HPV44	2/39	2.59 (0.59–11.44)	2.53 (0.55–11.51)	2/77	1.02 (0.23–4.52)	0.95 (0.21–4.23)
	HPV54	1/18	2.13 (0.28–16.37)	4.67 (0.58–37.89)	1/19	2.72 (0.34–21.54)	3.36 (0.41–27.40)
	HPV69/71^e^	0/8			2/40	3.41 (0.74–15.62)	3.38 (0.71–16.01)
	HPV74	4/36	6.02 (1.90–19.04)*	5.27 (1.64–17.00)*	1/64	0.77 (0.10–5.88)	0.69 (0.09–5.33)
	ART >2 years	8/293	1	1	7/264	1	1
**ART status**	ART ≤2 years	3/176	0.61 (0.16–2.32)	0.59 (0.15–2.31)	5/195	1.00 (0.32–3.16)	0.66 (0.19–2.33)
	ART-naive	5/177	1.07 (0.31–3.72)	0.99 (0.28–3.48)	4/228	1.21 (0.35–4.24)	1.17 (0.32–4.26)
	>500	4/253	1		4/260	1	
**Enrolment**	351–500	8/192	2.44 (0.73–8.20)		5/209	1.35 (0.36–5.08)	
**CD4+ count**	201–350	3/136	1.62 (0.36–7.26)		4/158	1.85 (0.46–7.47)	
**(cells/μL)**	<200	1/59	1.22 (0.14–10.88)		4/59	3.00 (0.66–13.61)	
	**Enrolment CD4+ count (cells/μL)**						
	>500	1/62	1		0/6	1	
**ART-naive**	351–500	2/54	2.19 (0.19–25.36)		1/50	0.12 (0.01–2.64)	
	201–350	2/45	4.71 (0.41–54.47)		1/75	0.62 (0.05–7.57)	
	<200	0/15			2/97		
	**HIV-1 viral suppression**						
	<1000 copies/ml	9/411	1	1	11/374	1	1
	≥1000 copies/ml	2/58	1.60 (0.34–7.54)	1.85 (0.31–10.90)	1/78	0.42 (0.05–3.40)	0.38 (0.05–3.08)
	**HIV-1 viral detection**						
	≤40 copies/ml	7/366	1	1	4/153	1	1
**ART users**	>40 copies/ml	4/103	1.97 (0.56–6.68)	2.32 (0.60–8.90)	8/297	0.98 (0.30–3.28)	1.03 (0.30–3.38)
	**Enrolment CD4+ count (cells/μL)**						
	>500	3/191	1		2/63	1	
	351–500	6/138	2.54 (0.63–10.29)		4/134	2.45 (0.44–13.48)	
	201–350	1/90	0.81 (0.08–7.81)		3/107	2.80 (0.46–16.99)	
	<200	1/44	1.57 (0.16–15.12)		3/54	4.59 (0.76–27.89)	

Adjusted Hazard Ratio (aHR): For Model 1

^a^In Burkina Faso, associations with incident AGW were adjusted for age and number of sexual partners in the previous 3 months

^b^In South Africa, associations with incident AGW were adjusted for age and mycoplasma genitalium; For Model 2: same adjustment as Model 1, in addition to enrolment CD4+ count

^c^ Any LR-HPV = HPV6/11/40/43/44/54/70/69/71/74

^d^There were no observations for Type 11 and 40 in both countries

^e^INNOLiPA does not discriminate between HPV types 69 an d 71

*All factors with p<0.05 are in bold.

## Discussion

We found that the prevalence and incidence of LR-HPV infection in this population were high and consistent with other studies of WLHIV in sub-Saharan Africa.[7, 25, 26] We also found that infections with types 6 and 11 were strongly associated with both prevalent and incident (type 6 only) AGW, which confirms the literature.[7, 27] The prevalence of AGW in both countries is similar to what has been reported among HIV-1 seropositive female sex workers in Burkina Faso, [7] but the AGW incidence (BF = 2.47 and SA = 2.33 per 100 person-years) was almost 3-fold lower than the 7.14 per 100 person-years reported by Low et al.,[7] possibly owing to the repeated occupational exposure and lower proportion of WLHIV on ART (10%) in that population.

We found that low enrolment CD4+ cell count was associated with a higher prevalence and persistence of LR-HPV as well as a higher prevalence and incidence of AGW. This is consistent with previous studies reporting CD4+ count as a strong predictor of HPV infection and AGW development.[9, 10] A longer duration (>2 years) of ART was not protective *per se* against prevalent LR-HPV infection, incident LR-HPV infection and incident AGW, confirming other studies findings.[7, 11] This suggests that other co-factors such as adherence to ART, HIV-1 PVL suppression and immunological reconstitution (as measured by increases in CD4+ count) are also important for the control of HPV infection and progression to associated disease.

We also found that, as reported in previous studies, non-HIV related factors such as alcohol use, number of recent sexual partners, changes in vaginal flora and coinfection with other STIs were significant co-factors for LR-HPV infection and AGW.[6, 7] In contrast to reports from previous studies, we did not find significant association between smoking or hormonal contraception with LR-HPV infection or AGW.[6, 28, 29] It was also surprising to note that LR-HPV prevalence was high among women who reported consistent condom use. The reason why infection prevalence was high among women who reported consistent condom use is not known. However, this could be related to social desirability bias which meant that women with high risk sexual behaviours reported high condom use.

Limitations of the study include the absence of HIV-1 PVL monitoring, in particular at endline and the possible subjective nature of clinical examination for AGW detection without histological confirmation leading to possible misclassification. Cervical LR-HPV infection rather than vaginal/vulval LR-HPV infection was measured which would have been more appropriate given the frequent external location of AGW. This can lead to difficulties in ascribing causality. However, cervical HPV infection has been used previously in determining associations with AGW.[7] Despite these limitations, this study had a number of strengths, including its longitudinal design, availability of AGW data at four time points and availability of genotyping data at both enrolment and endline visits. More importantly, the study contributes data on the epidemiology of LR-HPV infection and AGW from two sub-Saharan African countries with different HIV epidemics and this allows the findings to be extended to a range of countries and settings in the region.

## Conclusions

This study shows that WLHIV in sub-Saharan Africa are at high risk of LR-HPV infections and AGW. AGW were associated with HPV 6/11 and these findings support the argument for the use of quadri- or nano-valent HPV vaccines for the prevention of AGW among WLHIV. Effective use of ART with immunological reconstitution could also help control the burden of AGW among WLHIV in the region.

## Supporting information

S1 Table Effect of HIV-related factors on LR-HPV persistence, using infections as unit of measure. Adjusted Odds Ratio (aOR) using generalised estimating equations: ^a^In BF, associations with LR-HPV were adjusted for alcohol use, condom use and number of pregnancies; ^b^In SA, associations with LR-HPV were adjusted for number of regular sex partners, number of pregnancies and number of lifetime sex partners; For Model 2: same adjustment as Model 1, in addition to enrolment CD4+ count; ^c^ Of the 156 infections in Burkina Faso at enrolment (Table 3), 5 were excluded from analysis as they were ART-naïve but with undetectable viral load and similarly of the 240 infections at enrolment in South Africa (Table 3), 2 were excluded from the analysis; ^**d**^ART use was defined as being on ART at both enrolment and endline; *All factors with p<0.05 are in bold.
(DOC)

S1 File Full data set and codebook. (XLS)
