# Supplementary material for: Prevalence, incidence and correlates of low risk HPV infection and anogenital warts in a cohort of women living with HIV in Burkina Faso and South Africa
Source: PLoS One. 2018 May 1;13(5):e0196018. doi: 10.1371/journal.pone.0196018 (PMC5929550; doi:10.1371/journal.pone.0196018)
Supplement: S1 Table — Adjusted Odds Ratio (aOR) using generalised estimating equations: aIn BF, associations with LR-HPV were adjusted for alcohol use, condom use and number of pregnancies; bIn SA, associations with LR-HPV were adjusted for number of regular sex partners, number of pregnancies and number of lifetime sex partners; For Model 2: same adjustment as Model 1, in addition to enrolment CD4+ count; c Of the 156 infections in Burkina Faso at enrolment (Table 3), 5 were excluded from analysis as they were ART-naïve but with undetectable viral load and similarly of the 240 infections at enrolment in South Africa (Table 3), 2 were excluded from the analysis; dART use was defined as being on ART at both enrolment and endline; *All factors with p<0.05 are in bold. (DOC) [file pone.0196018.s001.doc]

**S1** Table. Effect of HIV-related factors on LR-HPV persistence, using infections as unit of measure

|  |  |  |  | **Burkina Faso^a^** |  |  |  | **South Africa^b^** |  |
| --- | --- | --- | --- | --- | --- | --- | --- | --- | --- |
|  |  |  |  | **N=151^c^** |  |  |  | **N=238^c^** |  |
|  |  |  |  | **Model 1** | **Model 2** |  |  | **Model 1** | **Model 2** |
|  |  | **N** | **n (%)** | **aOR (95% CI)** | **aOR (95% CI)** | **N** | **n (%)** | **aOR (95% CI)** | **aOR (95% CI)** |
| **ART status at baseline** | |  |  |  |  |  |  |  |  |
| ART >2 years | | 76 | 24 (31.6) | 1 | 1 | 85 | 24 (28.2) | 1 | 1 |
| ART ≤2 years | | 40 | 15 (37.5) | 1.20 (0.53-2.72) | 1.19 (0.53-2.70) | 77 | 25 (32.5) | 1.14 (0.87-1.50) | 1.02 (0.70-1.51) |
| ART-naive | | 35 | 10 (28.6) | 0.82 (0.42-1.60) | 0.72 (0.40-1.30) | 76 | 23 (30.3) | 1.38 (0.70-2.71) | 1.48 (0.73-2.99) |
| **Enrolment CD4+ count (cells/μL)** | |  |  |  |  |  |  |  |  |
| 500 | | 50 | 15 (30.0) | 1 | **-** | 87 | 25 (28.7) | 1 | **-** |
| 351-500 | | 43 | 14 (32.6) | 1.19 (0.72-1.98) | **-** | 73 | 20 (27.4) | 0.72 (0.34-1.49) | **-** |
| 200-350 | | 34 | 10 (29.4) | 1.02 (0.72-1.43) | **-** | 57 | 19 (33.3) | 1.18 (0.53-2.60) | **-** |
| <200 | | 24 | 10 (41.7) | 2.12 (0.73-6.36) | **-** | 21 | 8 (38.1) | 1.76 (0.58-5.32) | **-** |
| ***Among ART-naïve:*** | |  |  |  |  |  |  |  |  |
| **Enrolment CD4+ count (cells/μL)** | |  |  |  |  |  |  |  |  |
| >500 | | 5 | 0 (0.0) | **-** | **-** | 33 | 11 (33.3) | 1.00 | **-** |
| 351-500 | | 10 | 2 (20.0) | **-** | **-** | 26 | 8 (30.8) | 0.89 (0.24-3.33) | **-** |
| 200-350 | | 10 | 5 (50.0) | **-** | **-** | 15 | 4 (26.7) | 1.61 (0.37-4.59) | **-** |
| <200 | | 10 | 3 (30.0) | **-** | **-** | 2 | 0 (0.0) | - | **-** |
| ***Among ART users*^d^*:*** | |  |  |  |  |  |  |  |  |
| **Enrolment CD4+ count (cells/μL)** | |  |  |  |  |  |  |  |  |
| >500 | | 45 | 15 (33.3) | 1 | **-** | 54 | 14 (25.9) | 1 | **-** |
| 351-500 | | 33 | 12 (36.4) | 1.18 (0.66-2.13) | **-** | 47 | 12 (25.5) | 0.65 (0.31-1.38) | **-** |
| 200-350 | | 24 | 5 (20.8) | 0.55 (0.21-1.49) | **-** | 42 | 15 (35.7) | 1.39 (0.42-4.61) | **-** |
| <200 | | 14 | 7 (50.0) | 3.21 (0.90-11.40) | **-** | 19 | 8 (42.1) | **3.33 (0.96-11.55)*** | **-** |
| **HIV-1 viral suppression** | |  |  |  |  |  |  |  |  |
| <1000 copies/ml | | 95 | 32 (33.7) | 1 | 1 | 133 | 37 (27.8) | 1 | 1 |
| ≥1000 copies/ml | | 14 | 4 (28.6) | 0.95 (0.15-5.93) | 0.49 (0.04-5.62) | 29 | 12 (41.4) | 2.82 (0.60-13.38) | 2.77 (0.46-16.53) |
| **HIV viral detection^*^** | |  |  |  |  |  |  |  |  |
| ≤40 copies/ml | | 85 | 31 (36.5) | 1 | 1 | 48 | 15 (31.3) | 1 | 1 |
| >40 copies/ml | | 24 | 5 (20.8) | 0.44 (0.06-3.06) | 0.17 (0.01-4.26) | 114 | 34 (29.8) | 1.01 (0.27-3.69) | 0.90 (0.25-3.20) |

Adjusted Odds Ratio (aOR) using generalised estimating equations: ^a^In BF, associations with LR-HPV were adjusted for alcohol use, condom use and number of pregnancies; ^b^In SA, associations with LR-HPV were adjusted for number of regular sex partners, number of pregnancies and number of lifetime sex partners; For Model 2: same adjustment as Model 1, in addition to enrolment CD4+ count; ^c^ Of the 156 infections in Burkina Faso at enrolment (table 3), 5 were excluded from analysis as they were ART-naïve but with undetectable viral load and similarly of the 240 infections at enrolment in South Africa (table 3), 2 were excluded from the analysis; **^d^**ART use was defined as being on ART at both enrolment and endline; *All factors with p<0.05 are in bold.
